# Commentary on: “A Multi-Institutional Experience in Pediatric High Grade Glioma”

**DOI:** 10.3389/fonc.2015.00088

**Published:** 2015-04-08

**Authors:** Maria Caffo

**Affiliations:** ^1^Department of Neuroscience, School of Medicine, University of Messina, Messina, Italy

**Keywords:** pediatric high-grade glioma, chemotherapy, pediatric cancer, radiotherapy, molecular target therapy

This multi-institutional retrospective study evaluates the association of clinical, pathological, and treatment characteristics with their outcomes. Their results show total resection and receiving chemotherapy adjuvant to radiation or chemoradiation are most strongly related with improved progression-free survival (PFS) and overall survival. For higher risk incompletely resected patients, temozolomide (TMZ) use and treatment intensification with concurrent chemoradiation, adjuvant chemotherapy, and higher radiation dose were associated with improved outcomes. It is a well written and conducted paper about a remarkable challenge to neurosurgeon and pediatric oncologists. Little progress has been made in the outcome of these tumors in the last four decades. Outcome remains dismal with <20% of patients surviving (1). The degree of surgical resection is one of the most important clinical prognostic factors known in children with supratentorial high-grade gliomas (HGGs), independent of location, histology, and age as the authors show (2).

Chemotherapy was first introduced into the treatment schema for children with newly diagnosed HGG in 1970s. One the first randomized study was CCG-943 trial, in which children with newly diagnosed HGG were randomized to receive either focal radiation therapy alone to a dose of 54 Gy or the same radiotherapy with a combination of concomitant and maintenance chemotherapy. Patients randomized to receive chemotherapy were given weekly vincristine during radiation followed by eight maintenance chemotherapy cycles consisting of prednisone, lomustine, and vincristine (PCV) each given approximately 6 weeks apart (3). This important study showed that treatment with chemotherapy prolonged the survival and event-free survival. The Children’s Cancer Group (CCG) study-945 showed that children with HGG who underwent a surgical resection of 90% or greater had a PFS of 35 ± 7% as compared to a 5-year PFS of 17 ± 4% in patients who did not (4). The authors did not refer any benefit to the treatment of high-grade astrocytomas in children with eight-drugs in 1-day chemotherapy compared with CCNU, vincristine, and prednisone. Extent of tumor resection and histopathologic diagnosis were significant prognostic variables (4). The CCG-945 trial also looked at a variety of molecular and cytogenetic markers in an effort to better define prognostic variables in pediatric HGG.

Currently, the efficacy of chemotherapeutic compounds against pediatric brain tumors is unsatisfactory. Efficacy of TMZ has generally been found to be non-superior to other chemotherapeutic agents in pediatric HGG, and various studies in children have shown no benefit over conventional treatment (5). Cilengitide was well tolerated in pediatric HGG, yet had modest anti-tumor activity as a single agent (6). However, cilengitide in combination with radiation and chemotherapy, TMZ, has shown synergistic activity (7). Phase I or II studies of small molecules that target specific pathways or proteins in the cancer cells such as gefitinib (8), imatinib (9), cloretazine (10), tipifarnib (11), erlotinib (12), and nimotuzumab (13) disclosed no efficacy, despite occasional remarks of stable disease (1). Recently, fractionated schedules of drug administration using smaller doses than the maximum tolerated dose (metronomic schedules) have been reported that might enhance the antiangiogenic activity of some chemotherapeutic agents (14). Metronomic chemotherapy shows potential advantages in pediatric brain tumors treatment, including primary effect on the host cells of the tumor microenvironment, the possibility of greater long-term efficacy and tolerability than conventional cytotoxic therapy (14).

In recent years, important advances in the comprehension of the molecular characteristics of HGG in pediatric age have been made. Initial genomic studies of pediatric HGG disclosed significant differences compared to tumors from adult patients suggesting the existence of molecularly diverse subsets within pediatric cohorts. Data about the interactions between genetic alterations and changes in DNA methylation, histone modifications, chromatin remodeling, and gene expression contribute to explain pathogenesis of malignant gliomas. Adult HGG are characterized by IDH1, PTEN, and/or EGFR aberrations; whereas pediatric HGG often harbors PDGFR amplification. PDGFR amplification or overexpression represents the most common abnormality of HGG in pediatric patients (15). BRAF and CDKN2A mutations have been reported to characterize HGG in a subset of pediatric patients, although BRAF abnormalities are not as frequently as described in childhood low-grade glioma (15). Schwartzentruber et al. reported in a large cohort of gliomas of various grades and histologies H3F3A mutations to be prevalent in children and young adults affected by malignant gliomas (16). Mutations of genes H3F3A and HIST1H3B are epigenetic and play a role in chromatin remodeling (17). Mutations in IDH1 or IDH2 are exceptional in pediatric patients but occur in the majority of adult patients with HGG. A study of pediatric primary HGG from the Children’s Oncology Group observed IDH1 mutations in 7 of 43 tumors. Remarkably, all of these IDH1 mutations occurred in children ≥14 years old, with none occurring in younger children. No IDH2 mutations were observed (18). IDH1 mutations at codon 132 strongly differentiate adult secondary from primary glioblastoma, with frequencies of 85% compared with 5%. Paugh et al. sequenced IDH1 exon 4, containing codon 132, from 78 pediatric HGGs and 11 pediatric low-grade gliomas, and no codon 132 mutations were detected (15).

The recent genomic groundbreaking work added tremendously to our understanding of the mutational landscape of pediatric HGG, bringing to light new, potentially targetable pathways, and promise of more effective therapies. In light of these observations, new trials and emerging data about pediatric HGG could improve the outcome of HGG in children and young adults.

## Conflict of Interest Statement

The author declares that the research was conducted in the absence of any commercial or financial relationships that could be construed as a potential conflict of interest.

## References

[1] MacDonald TJ Aguilera D Kramm CM. Treatment of high-grade glioma in children and adolescents. Neuro Oncol (2011) 13:1049–58. 10.1093/neuonc/nor092 21784756 PMC3177659

[2] Adamski J Tabori U Bouffet E Advances in the management of paediatric high-grade glioma. Curr Oncol Rep (2014) 16:414 10.1007/s11912-014-0414-0 25410415

[3] Sposto R Ertel IJ Jenkin RD Boesel CP Venes JL Ortega JA The effectiveness of chemotherapy for treatment of high grade astrocytoma in children: results of a randomized trial. A report from the childrens cancer study Group. J Neurooncol (1989) 7:165–77. 10.1007/BF00165101 2550594

[4] Finlay JL Boyett JM Yates AJ Wisoff JH Milstein JM Geyer JR Randomized phase III trial in childhood high-grade astrocytoma comparing vincristine, lomustine, and prednisone with the eight-drugs-in-1-day regimen. Childrens cancer group. J Clin Oncol (1995) 13(1):112–23. 7799011 10.1200/JCO.1995.13.1.112

[5] Nicholson HS Kretschmar CS Krailo M Bernstein M Kadota R Fort D Phase 2 study of temozolomide in children and adolescents with recurrent central nervous system tumors: a report from the children’s oncology group. Cancer (2007) 110:1542–50. 10.1002/cncr.22961 17705175

[6] MacDonald TJ Vezina G Stewart CF Turner D Pierson CR Chen L Phase II study of cilengitide in the treatment of refractory or relapsed high-grade gliomas in children: a report from the children’s oncology group. Neuro Oncol (2013) 15(10):1438–44. 10.1093/neuonc/not058 24014381 PMC3779033

[7] Nabors LB Mikkelsen T Hegi ME Ye X Batchelor T Lesser G A safety run-in and randomized phase 2 study of cilengitide combined with chemoradiation for newly diagnosed glioblastoma (NABTT 0306). Cancer (2012) 118:5601–7. 10.1002/cncr.27585 22517399 PMC3423527

[8] Geyer JR Stewart CF Kocak M Broniscer A Phillips P Douglas JG A phase I and biology study of gefitinib and radiation in children with newly diagnosed brain stem gliomas or supratentorial malignant gliomas. Eur J Cancer (2010) 46:3287–93. 10.1016/j.ejca.2010.07.005 20708924 PMC2988095

[9] Pollack IF Jakacki RI Blaney SM Hancock ML Kieran MW Phillips P Phase I trial of imatinib in children with newly diagnosed brainstem and recurrent malignant gliomas: a pediatric brain tumor consortium report. Neuro Oncol (2007) 9:145–60. 10.1215/15228517-2006-031 17293590 PMC1871662

[10] Gururangan S Turner CD Stewart CF O’Shaughnessy M Kocak M. Phase I trial of VNP40101M (cloretazine) in children with recurrent brain tumors: a pediatric brain tumor consortium study. Clin Cancer Res (2008) 14(4):1124–30. 10.1158/1078-0432.CCR-07-4242 18281546

[11] Fouladi M Nicholson HS Zhou T Laningham F Helton KJ Holmes E A phase II study of the farnesyl transferase inhibitor, tipifarnib, in children with recurrent or progressive high-grade glioma, medulloblastoma/primitive neuroectodermal tumor, or brainstem glioma: a children’s oncology group study. Cancer (2007) 110:2535–41. 10.1002/cncr.23078 17932894

[12] Broniscer A Baker SJ Stewart CF Merchant TE Laningham FH Schaiquevich P Phase I and pharmacokinetic studies of erlotinib administered concurrently with radiotherapy for children, adolescents, and young adults with high-grade glioma. Clin Cancer Res (2009) 15:701–7. 10.1158/1078-0432.CCR-08-1923 19147777 PMC2629527

[13] Cabanas R Saurez G Rios M Alert J Reyes A Valdes J Treatment of children with high grade glioma with nimotuzumab: a 5-year institutional experience. MAbs (2013) 5(2):202–7 10.4161/mabs.22970 23575267 PMC3893230

[14] Robison NJ Campigotto F Chi SN Manley PE Turner CD Zimmerman MA A phase II trial of a multi-agent oral antiangiogenic (metronomic) regimen in children with recurrent or progressive cancer. Pediatr Blood Cancer (2014) 61:636–42. 10.1002/pbc.24794 24123865 PMC4285784

[15] Paugh BS Qu C Jones C Liu Z Adamowicz-Brice M Zhang J Integrated molecular genetic profiling of pediatric high-grade gliomas reveals key differences with the adult disease. J Clin Oncol (2010) 28(18):3061–8. 10.1200/JCO.2009.26.7252 20479398 PMC2903336

[16] Schwartzentruber J Korshunov A Liu XY Jones DT Pfaff E Jacob K Driver mutations in histone H3.3 and chromatin remodelling genes in paediatric glioblastoma. Nature (2012) 482(7384):226–31. 10.1038/nature10833 22286061

[17] Sturm D Bender S Jones DT Lichter P Grill J Becher O Paediatric and adult glioblastoma: multiform (epi)genomic culprits emerge. Nat Rev Cancer (2014) 14(2):92–107. 10.1038/nrc3655 24457416 PMC4003223

[18] Pollack IF Hamilton RL Sobol RW Nikiforova MN Lyons-Weiler MA LaFramboise WA IDH1 mutations are common in malignant gliomas arising in adolescents: a report from the children’s oncology group. Childs Nerv Syst (2011) 27:87–94. 10.1007/s00381-010-1264-1 20725730 PMC3014378

